# Epidemiology of *Pestivirus H* in Brazil and Its Control Implications

**DOI:** 10.3389/fvets.2021.693041

**Published:** 2021-07-23

**Authors:** Fernando V. Bauermann, Julia F. Ridpath

**Affiliations:** ^1^Department of Veterinary Pathobiology, College of Veterinary Medicine, Oklahoma State University (OSU), Stillwater, OK, United States; ^2^Ridpath Consulting, LLC, Gilbert, IA, United States

**Keywords:** atypical bovine pestivirus, control, diagnostic, HoBi-like virus, South America

## Abstract

Along with viruses in the *Pestivirus A* (*Bovine Viral Diarrhea Virus 1*, BVDV1) and *B* species (*Bovine Viral Diarrhea Virus 2*, BVDV2), members of the *Pestivirus H* are mainly cattle pathogens. Viruses belonging to the Pestivirus H group are known as HoBi-like pestiviruses (HoBiPev). Genetic and antigenic characterization suggest that HoBiPev are the most divergent pestiviruses identified in cattle to date. The phylogenetic analysis of HoBiPev results in at least five subgroups (a–e). Under natural or experimental conditions, calves infected with HoBiPev strains typically display mild upper respiratory signs, including nasal discharge and cough. Although BVDV1 and BVDV2 are widely distributed and reported in many South American countries, reports of HoBiPev in South America are mostly restricted to Brazil. Despite the endemicity and high prevalence of HoBiPev in Brazil, only HoBiPev-a was identified to date in Brazil. Unquestionably, HoBiPev strains in BVDV vaccine formulations are required to help curb HoBiPev spread in endemic regions. The current situation in Brazil, where at this point only HoBiPev-a seems present, provides a more significant opportunity to control these viruses with the use of a vaccine with a single HoBiPev subtype. Despite the lack of differentiation among bovine pestiviruses by current BVDV tests, the reduced genetic variability of HoBiPev in Brazil may allow reliable identification of cases within the region. On the other hand, introducing foreign ruminants, biologicals, and genetic material to South America, especially if it originated from other HoBiPev-endemic countries, should consider the risk of introducing divergent HoBiPev subtypes.

## Introduction

### *Pestivirus H* History Recap

The pestivirus genus within the family *Flaviviridae* underwent extensive taxonomic revisions in the past few years, leading to many of its members' classification or reclassification. For decades, only four viral species were officially recognized and historically known as Bovine Viral Diarrhea Virus 1 and 2 (BVDV1 and BVDV2), Classical Swine Fever Virus, and Border Disease Virus. These species are now classified as Pestivirus A, B, C, and D. Several pestiviruses described as “atypical” are currently officially recognized at the species level (1). The seven recently classified species are the Pestivirus E (Pronghorn pestivirus), Pestivirus F (Bungowannah virus), Pestivirus G (Giraffe pestivirus), Pestivirus H (HoBi-like pestivirus), Pestivirus I (Aydin-like pestivirus), Pestivirus J (rat pestivirus), and Pestivirus K (atypical porcine pestivirus) (2).

Among the new pestivirus species, *Pestivirus H* and *K* are likely the most widespread “new” species (3, 4). Both species have been reported in the American continent, Europe, and Asia (3, 4). Members of the *Pestivirus K* species were only described infecting pig (5), while *Pestivirus H* is mainly a bovine pathogen. However, natural infection with viruses from the *Pestivirus H* species has been described in small ruminant species in Asia and in water buffalos in Brazil and Argentina (6–10).

*Pestivirus H* was discovered in Germany in 2004 as a cell culture contaminant (11). At that time, the virus was designated as an atypical bovine pestivirus. The strain was named HoBi_D32/00, referencing the researchers, Horst and Birgit, who isolated and characterized the virus. The virus's origin was traced to a Brazilian fetal bovine serum (FBS) used as a media supplement for cell culture in that laboratory (11). The HoBi_D32/00 virus was first described as a putative new pestivirus species. Initially, group members were called atypical bovine pestivirus, BVDV3, or HoBi-like pestiviruses (HoBiPev) (12).

Studies comparing HoBiPev genetic and antigenic characteristics to those observed between BVDV1 (*Pestivirus A*) and BVDV2 (*Pestivirus B*) demonstrated that HoBiPev was the most divergent ruminant pestivirus identified to date (11, 13). The characterization of additional HoBiPev isolates strengthened the overall understanding of the genetic and antigenic differences between *Pestivirus H, A*, and *B* species and their implications toward the diagnostic and control of ruminant pestiviruses (8, 14, 15). Recent reports of genetic characterization of HoBiPev suggest that at least five subtypes (a–e) may exist (16).

### Geographic Distribution of HoBiPev

Almost concurrently with the first reporting of HoBi_D32/00 in Germany, between 2003 and 2004, a HoBiPev was identified in a dairy herd in Thailand without any history of disease (17). The isolate was denominated Khonkaen, and phylogenetic analysis indicated that while it grouped with HoBi_D32/00, it was significantly divergent from the South American isolate, providing the first evidence of HoBiPev subtypes (14). The marked genetic differences between the first two isolates of HoBiPev gave a strong indication that the virus was present and evolving independently in both continents for a period of time before its identification. Following the first description and tracking of HoBiPev to an FBS that originated in Brazil, the suggestion of a novel pestivirus species circulating in South America sparked curiosity among researchers in that region, and quickly, additional cases of HoBiPev were reported, including cases previously misclassified as BVDV (3, 18–21). Currently, it has been demonstrated that HoBiPev is present in major beef- and dairy-producing areas in Brazil (south and central regions), although its true prevalence is unknown (18, 19, 22). Interestingly, in the north-eastern part of the country, HoBiPev is the most prevalent ruminant pestivirus (23). Notably, all 17 pestivirus-positive samples identified by testing 16,621 bovine serum samples belonged to the HoBiPev group (23). Interestingly, the study also tested 2,672 serum samples from small ruminants in the same region with no HoBiPev being identified (23).

In 2010, the virus was identified in Italy (8, 24). The identification of HoBiPev in Europe was a significant event, and it was theorized that additional cases would continue to arise in Europe. However, the outbreak was restricted to a single farm. Notably, after 3 years, the virus re-emerged in the same farm causing abortions (25). The origin of the virus related to the outbreak in Europe was never fully understood. However, the isolates' genetic characterization demonstrated a closer relationship to South American isolates than the Thai isolate. Analyses of archival samples from Italian herds dating back to 2005 revealed the circulation of HoBiPev in the country as early as 2007, mostly associated with samples of cattle with respiratory disease (26). Additionally, in 2019, HoBiPev was reported in Turkey in a blood sample collected between 2016 and 2017 that had previously been identified as BVDV positive. Genetic characterization classified the sequence within the HoBiPev group (27).

HoBiPev has been described in countries in the Indian subcontinent, particularly in India and Bangladesh (9, 10, 16). Those studies revealed significant genetic diversity in the isolates from that region, suggesting circulation and evolution of HoBiPev in ruminants for a prolonged time rather than a recent introduction event (9, 10). The description of the pestivirus surveillance in India suggested that HoBiPev was the most prevalent ruminant pestivirus in the studied regions based on the test of 1,049 bovine serum samples. Remarkably, out of the 20 samples positive for pestivirus, 19 were phylogenetically grouped with HoBiPev strains (10).

HoBiPev has been detected in goats in China, although without a clear association with disease (7). Recently, in 2017, an outbreak of HoBiPev with high mortality rates in beef cattle around 7–8 months old was described in China (28). The affected herd was composed of 140 animals, and the mortality rate was over 50% (78 animals). Despite unique amino acid changes identified in the E2 and Npro region of the Chinese HoBiPev-a, the possible correlation with virulence is unknown (28). The description of HoBiPev involved in severe disease cases remains a matter of concern. Further investigation is required to address whether this isolate presents an increased virulence phenotype or whether the severe clinical presentation was a consequence of multi-factorial conditions.

In addition to identifying naturally infected ruminants with HoBiPev in Brazil and European and Asian countries, there is serological evidence of HoBiPev circulation in water buffalos in Argentina (6). HoBiPev was also detected in North American, Argentinian, and Australian lots of commercial FBS (29, 30). Despite the report of contaminated FBS in lots labeled as originated from Mexico and the USA, there was no evidence for the circulation of HoBiPev during comprehensive surveillance of about 2,000 bovine serum samples representing all the mainland US states (31). Additionally, the testing of FBS lots originated and packed in the US producing plants supported that the US remained free of HoBiPev (32). Similarly, extensive characterization of pestiviruses in Mexico failed to identify HoBiPev (33, 34).

### HoBiPev Infection—Clinical Remarks

Under natural or experimental conditions, calves infected with HoBiPev strains typically display mild (if any) upper respiratory signs (10, 17, 35–37). Despite the relatively low pathogenicity of HoBiPev strains identified to date, calves experimentally infected with HoBiPev strains exhibited a significant level of thymus atrophy, similar to those identified in typical virulent BVDV strains (38, 39).

More prominent disease cases involving HoBiPev were reported in Italy and China, including severe respiratory signs and diarrhea (7, 8). The high level of genome homology of the viruses isolated from those animals undergoing severe disease compared to other isolates from South America may suggest that disease severity was a consequence of different factors, including stressful conditions, other pathogens, and herd genetics. However, the emergence of highly virulent ruminant pestiviruses is a well-known phenomenon (40, 41) and eventually may occur with HoBiPev.

A key ruminant pestivirus characteristic is its tropism for fetal tissues and the establishment of persistent infection (42). Such persistently infected (PI) animals shed the virus to the environment continuously throughout their lifetimes (42, 43). We previously demonstrated that both South American and Italian non-cytopathic HoBiPev strains infected the fetus in 100% of inoculated heifers (44). The presence of HoBiPev PI animals has been reported in the field (16, 20, 22, 45). Like BVDV PIs, HoBiPev PIs also may present with the highly lethal mucosal disease (MD) syndrome (16, 20, 22, 45, 46). The MD syndrome is characterized by necrotic and erosive lesions on the gastrointestinal tract mucosa, associated with other symptoms, including enteric and respiratory signs (47). Four cases of HoBiPev-infected animals displaying MD-like symptoms have been reported (16, 20, 22, 45). All of the reports to date describe the presence of classical pathological findings comparable to BVDV MD cases. Two of these reports were from cases in Brazil. One in the north-eastern part of the country is described in a calf (20), whereas the second is an outbreak of MD-like symptoms in a case reported in the country's central west region (22).

### Control and Diagnostic

Specific control measures for HoBiPev, including commercial vaccines, are unavailable. Due to the antigenic similarities that HoBiPev shares with BVDV1 and BVDV2, a study evaluating the potential cross-protection was conducted. Virus neutralization assays with HoBiPev-a, BVDV1-b, BVDV2-a, and border disease virus were performed using the serum of cattle immunized by either a MLV or killed vaccine containing both BVDV species (48). It was found that BVDV-vaccinated cattle had low cross-neutralizing antibody levels against HoBiPev-a with more than 90% of the animals demonstrating antibody-neutralizing titers to HoBiPev lower than 20 (44).

Subsequently, we demonstrated limited fetal protection to HoBiPev in cows that generated either a BVDV1 or BVDV2 PI in a previous gestation (49). HoBiPev was identified in the fetuses of 90% of infected cows, despite the cow's high level of neutralizing antibodies against BVDV1 or BVDV2 (49). Both of the studies, *in vitro* and *in vivo*, clearly demonstrated that robust immunity to either BVDV1 or 2 would have limited effect controlling the spread of HoBiPev in the event of introduction into a naïve population.

The antigenic variability among isolates belonging to the same pestivirus subtypes is described (50). Not surprisingly, the characterization of HoBiPev-a isolates in Brazil revealed significant antigenic diversity in the E2 protein, which hosts the major epitopes targeted by neutralizing antibodies (13, 19). The study conducted using eight HoBiPev-a isolates revealed that despite the conserved 5-UTR regions and clustering close together in the HoBiPev-a branch, the strains demonstrated significant antigenic diversity assessed by monoclonal antibody (MAb) panel and the level of neutralizing antibodies (19). Using 27 MAbs produced for BVDV, the reactivity of HoBiPev isolates ranged from 5 to 13 MAbs. As expected, it was verified by variation in the neutralization level of the antiserum among the different HoBiPev subtypes. There were higher cross neutralizing levels among HoBiPev-a isolates compared to both BVDV1 and 2 strains with the exception of one HoBiPev-a strain used in the study. The antiserum raised against seven HoBiPev strains had a limited neutralizing effect on the HoBiPev-a strain SV478/07, with a neutralizing level comparable or lower to the BVDV2 neutralizing level (19). The opposite was also true, the ability for the antiserum raised against SV478/07 had a limited neutralization effect on the other seven HoBiPev-a strains (19).

Based on the lack of specific control measures to HoBiPev, diagnostic testing is critical to avoid introduction of HoBiPev or its subtypes into free regions. The ability to quickly identify HoBiPev may also have a critical role in curbing viral spread. Despite its importance, specific commercial testing for HoBiPev is not available. The commercial RT-qPCR tests virotype BVDV RT-PCR Kit (Qiagen, Labor Diagnostik Leipzig GmbH, Leipzig, Germany) and VetMAX-Gold bovine virus diarrhea RNA test kit (Applied Biosystems, Life Technologies, Austin, TX, USA), designed for BVDV, demonstrated suitability in detecting positive samples from experimentally generated HoBiPev PI calves (44). The calves were harboring either the HoBi D32/00 or an Italian isolate, belonging to the subtype HoBiPev-a. Both tests accurately detected all PI animals using serum or buffy coat samples collected at day of birth (44). However, during the study, the virotype assay detected all tested samples, whereas VetMAX correctly identified about 85% of the positive samples (44). In a follow-up study conducted in India, expanding the understanding of these commercial BVDV kits in detecting divergent HoBiPev subtypes, samples spiked with the Asian HoBiPev types c and d were tested (51). The study demonstrated that virotype had higher sensitivity in detecting dilutions of these divergent subtypes than VetMAX-Gold. The virotype detection limits for HoBiPev-c and -d were 10^0.6^ TCID_50_/ml and 10^0.3^ TCID_50_/ml, while for VetMAX-Gold were 10^0.6^ TCID_50_/ml and 10^2.3^ TCID_50_/ml. However, virotype sensitivity to HoBiPev-c and -d was decreased compared to HoBiPev-a detection level (51).

Not surprisingly, the increased genetic variability of Asian HoBiPev led to increased antigenic diversity. The commercial ELISA test (IDEXX BVDV Ag/Serum Plus, IDEXX, Westbrook, ME, USA), which is based on detecting the Erns protein, mostly excels in detecting samples with HoBiPev-a (44). Although there is evidence that some HoBiPev-a strains may not be detected by this kit (15). The same kit had limited success in detecting samples with HoBiPev-c and -d, with detection limits of 10^4.6^ TCID_50_/ml and 10^6.3^ TCID_50_/ml (51). Other ELISA kits based on the NS3 protein typically demonstrated low sensitivity to HoBiPev-a, and the kit INgezim BVD DAS (Ingenasa, Madrid, Spain) completely failed in detecting the divergent subtypes c and d (13, 51).

## Discussion

Currently, there is no nationwide eradication program for BVDV in South America, and the BVDV control is entirely voluntary at the farm or regional level. In addition, vaccination use may be restricted due to its costs, and testing may be limited to commercial ELISA kits that do not identify the specific pestivirus strain. All these factors hamper a comprehensive understanding of the true epidemiology of ruminant pestiviruses in the region. Despite these challenges, many countries in South America have reported BVDV types 1 and 2 and multiple subtypes within those viruses (18, 52–59). Other than Brazil, Argentina is the only South American country that has documented the presence of HoBiPev. The testing of fetal bovine serum lots from Argentina identified HoBiPev-a in four lots (30). Also, in Argentina, HoBiPev circulation was evidenced by the serum conversion of water buffalos to HoBiPev (6).

Considering that Brazil borders 10 countries or territories in South America, it is likely that either HoBiPev has disseminated to additional regions or there is an imminent risk of its spread. Despite the high prevalence of HoBiPev in cattle herds in Brazil, with strong evidence for it being the most prevalent ruminant pestivirus in at least the north-eastern region, the description of infection in other species in South America is restricted to water buffalos (12). Despite the testing of thousands of small ruminant samples (sheep and goats) from north-eastern Brazil, no positive sample was identified and no other report of HoBiPev in small ruminants in South America is available to date. However, it should be noted that there is a lack of systematic testing of small ruminants, so that failure to detect may be associated with failure to test. The strain identified in small ruminants in China is genetically divergent from typical HoBiPev-a, and specific mutations may have led to the increased tropism to small ruminants. Further studies are required to identify the susceptibility of ruminant species to the different HoBiPev subtypes.

Independently of the HoBiPev subtype, most acute infections are clinically indistinguishable from typical, uncomplicated BVDV1 and BVDV2 infections. The descriptions of MD-like disease associated with HoBiPev infections in different parts of the world suggest that highly fatal forms of HoBiPev infection do exist. Typically, most of the PI animals die at an early age, usually within the first 6 months of life (42). The description of MD in South American and Italy, all involving HoBiPev-a, follows the scenario observed with BVDV mucosal disease, with the description of clinical signs in calves (20, 22, 45). However, the recent description of MD-like cases in India diverges from this typical scenario (16). Nine cases resembling MD were received in a veterinary hospital between 2018 and 2019 (16). Most of the animals with MD-like symptoms were between 2 and 4 years old. In addition to the unusual animal age for the development of MD, phylogenetic analyses demonstrated that one animal was harboring HoBiPev-d, whereas the remaining eight animals were harboring a putative new HoBiPev-e (16). It remains unclear if the atypical characteristics of these cases in India correlate to the divergent HoBiPev-d and -e subtypes circulating in the country or if it is related to the biased sampling collection method.

The genetic and antigenic diversity of pestiviruses is also a well-known nemesis of vaccine design. Whereas, the benefits of using vaccines with partial protection are debatable from the standpoint of accelerating virus divergence, it may help decrease the emergence of PI animals (60). Based on the antigenic characteristics of HoBiPev, assessed by monoclonal antibody panels, HoBiPev-a isolates demonstrated common epitopes with both BVDV1 and BVDV2 strains within the E2 protein (11, 13, 19). However, both *in vivo* and *in vitro* studies suggested low to no cross-protection. In the long-term, countries in South America or Asia using BVDV vaccines may provide a favorable scenario for HoBiPev becoming the most common ruminant pestiviruses in additional regions.

It is untested whether HoBiPev strains in BVDV vaccine formulations are required to help curb HoBiPev spread. The situation in Brazil and Argentina, where only HoBiPev-a was identified, provides a better opportunity to control these viruses with the addition of a single subtype in the vaccine compared to the other regions in the world.

Despite the critical need for specific HoBiPev diagnostic, no commercial test is available. Pestivirus-free regions and BVDV endemic regions will certainly benefit from commercially available kits with the capacity to differentiate BVDV from HoBiPev. The discovery of divergent HoBiPev subtypes also questions the efficacy of in-house tests previously designed for HoBiPev-a detection (32, 51, 61). The continuous use of BVDV tests with limited sensitivity for HoBiPev could allow a “silent” introduction and dissemination of these viruses into BVDV-free or -endemic regions. Despite the lack of differentiation of the BVDV tests, the reduced genetic variability of HoBiPev currently circulating in Brazil may allow for reliable identification of cases within the region. However, introducing foreign ruminants, biologicals, and genetic material to South America, primarily if originated in Asia, should consider the possible presence of divergent HoBiPev subtypes and the risk of introduction and spread in South American cattle herds.

## Author Contributions

FB and JR conceived and wrote the article. All authors contributed to the article and approved the submitted version.

## Conflict of Interest

JR was employed by the company Ridpath Consulting, LLC. The remaining author declares that the research was conducted in the absence of any commercial or financial relationships that could be construed as a potential conflict of interest.

## Publisher's Note

All claims expressed in this article are solely those of the authors and do not necessarily represent those of their affiliated organizations, or those of the publisher, the editors and the reviewers. Any product that may be evaluated in this article, or claim that may be made by its manufacturer, is not guaranteed or endorsed by the publisher.
